# *Anaplasma phagocytophilum* in sheep and goats in central and southeastern China

**DOI:** 10.1186/s13071-016-1880-z

**Published:** 2016-11-21

**Authors:** Jifei Yang, Zhijie Liu, Qingli Niu, Junlong Liu, Rong Han, Guiquan Guan, Youquan Li, Guangyuan Liu, Jianxun Luo, Hong Yin

**Affiliations:** 1 State Key Laboratory of Veterinary Etiological Biology, Key Laboratory of Veterinary Parasitology of Gansu Province, Lanzhou Veterinary Research Institute, Chinese Academy of Agricultural Sciences, Xujiaping 1, Lanzhou, Gansu 730046 People’s Republic of China; 2 Jiangsu Co-innovation Center for Prevention and Control of Important Animal Infectious Diseases and Zoonoses, Yangzhou, 225009 People’s Republic of China

**Keywords:** *Anaplasma phagocytophilum*, 16S rRNA gene, *groEL* gene, Ecotypes, Sheep, Goats, Novel *Anaplasma* species, China

## Abstract

**Background:**

*Anaplasma phagocytophilum* is wide spread throughout the world and impacts both human and animal health. Several distinct ecological clusters and ecotypes of the agent have been established on the basis of various genetic loci. However, information on the genetic variability of *A. phagocytophilum* isolates in China represents a gap in knowledge. The objective of this study was to determine the prevalence and genetic characterization of *A. phagocytophilum* in small ruminants in central and southeastern China.

**Methods:**

The presence of *A. phagocytophilum* was determined in 421 blood samples collected from small ruminants by PCR. Positive samples were genetically characterized based on 16S rRNA and *groEL* genes. Statistical analyses were conducted to identify ecotypes of *A. phagocytophilum* strains, to assess their host range and zoonotic potential.

**Results:**

Out of 421 sampled small ruminants, 106 (25.2%) were positive for *A. phagocytophilum*. The positive rate was higher in sheep (35.1%, 40/114) than in goats (26.4%, 66/307) (*P* < 0.05). Sequence analyses revealed that the isolates identified in this study were placed on two separate clades, indicating that two 16S rRNA variants of *A. phagocytophilum* were circulating in small ruminants in China. However, analysis using obtained *groEL* sequences in this study formed one cluster, which was separate from other known ecotypes reported in Europe. In addition, a novel *Anaplasma* sp. was identified and closely related to an isolate previously reported in *Hyalomma asiaticum*, which clustered independently from all recognized *Anaplasma* species.

**Conclusions:**

A molecular survey of *A. phagocytophilum* was conducted in sheep and goats from ten provinces in central and southeastern China. Two 16S rRNA variants and a new ecotype of *A. phagocytophilum* were identified in small ruminants in China. Moreover, a potential novel *Anaplasma* species was reported in goats. Our findings provide additional information on the complexity of *A. phagocytophilum* in terms of genetic diversity in China.

**Electronic supplementary material:**

The online version of this article (doi:10.1186/s13071-016-1880-z) contains supplementary material, which is available to authorized users.

## Background

*Anaplasma phagocytophilum* is an obligate intracellular bacterium that replicates mainly in neutrophils of humans and animals [1]. It is thought to be maintained primarily in an enzootic cycle between *Ixodes* ticks and vertebrate hosts and may cause human, canine, and equine granulocytic anaplasmosis and tick-borne fever in ruminants [2]. In addition, *A. phagocytophilum* infection has been reported in a variety of wild and domestic animals, especially in rodents and wild deer [3]. In China, the first suspected human case was described in Anhui Province in 2006 [4]; since then, an increasing number of HGA cases have been recorded in six cities and provinces [5].

The survival and replication of *A. phagocytophilum* in different tick species and a wide range of vertebrate hosts may lead to high genetic variability of the agent, as revealed by analysis of various genetic loci, including 16S rRNA, heat-shock operon (*groEL*), *ankA* and genes of major surface proteins [3, 6–10]. Recently, the *A. phagocytophilum* variants characterized based on the 16S rRNA gene have been typed extensively on the basis of the *groEL* gene in ticks and animals, and several lineages and ecotypes with different pathogenic abilities were reported in both European countries and USA [3, 8, 11, 12]. The molecular characterization of *A. phagocytophilum* strains in China was also reported, and several 16S rRNA variants were identified in ticks, small mammals and ungulates [5, 13–16]. However, it is unclear at this stage how the Chinese *A. phagocytophilum* isolates segregate into lineages or ecotypes and whether these isolates genetically differ from those pathogenic strains reported in Europe and in USA. In this study, the presence of *A. phagocytophilum* in small ruminants was investigated. The isolates of *A. phagocytophilum* were characterized by sequence analysis of the 16S rRNA and *groEL* genes and compared them to the strains reported earlier.

## Methods

### Study sites and collection of specimens

The survey was performed from March to September during the peak season of tick activities between 2011 and 2015 in rural areas of ten provinces containing 17 counties in central and southeastern China (Table 2). Two to three sites were selected for sampling in each county. EDTA whole-blood samples were taken randomly from the jugular vein of 421 asymptomatic small ruminants (sheep, *n* = 114; goats, *n* = 307) and collected in a sterile tube. Total DNA was extracted from 300 μl of blood using the Gentra Puregene Blood Kit (Qiagen, Beijing, China) according to the manufacturer’s instructions.

### PCR reactions

*Anaplasma phagocytophilum* DNA was detected by nested PCR for the amplification of the 16S rRNA gene as previously described [17, 18]. *Anaplasma* and *Ehrlichia* genus-specific primers EE1 and EE2 were used for primary reactions, and species-specific primers SSAP2f and SSAP2r were used for nested reactions (Table 1). The length of the nested PCR fragments was 641 base pairs. Positive samples were selected randomly and used for the amplification of the *groEL* gene [19]. The partial *groEL* gene sequences (574 bp) were amplified using EphplgroEL(569)F and EphplgroEL(1193)R primers for primary reactions and EphplgroEL(569)F and EphgroEL(1142)R primers for semi-nested reactions (Table 1) [19]. The reaction was performed in an automatic thermocycler (Bio-Rad) with a total volume of 25 μl containing 2.5 μl of 10× PCR buffer (Mg^2+^ Plus), 2.0 μl of each dNTP at 2.5 mM, 1.25 U of *Taq* DNA polymerase (TaKaRa, Dalian, China), 2.0 μl of template DNA, 1.0 μl of each primer (10 pmol), and 16.25 μl of distilled water. Genomic DNA extracted from the whole blood of sheep infected with *A. phagocytophilum* (GenBank accession no. JN558811) was used as the positive control, and sterile water was used as the blank control for each run. Cycling conditions for PCR amplification were: 4 min of denaturation at 94 °C, 35 cycles at 94 °C for 1 min, annealing for 45 s (annealing temperatures of primers are listed in Table 1), and 72 °C for 45 s- 1.5 min (dependent on the target genes), with a final extension step at 72 °C for 10 min. Amplified fragments were run on a 1.0% agarose gel following electrophoresis, staining with ethidium bromide, and then visualized by UV transillumination.

**Table 1:** Primers and PCR amplification conditions

Target gene	Primer name	Primer sequence (5’-3’)	Annealing temperature (°C)	Amplicon size (bp)	Reference
16S rRNA	EE-1	TCCTGGCTCAGAACGAACGCTGGCGGC	55	1433	[17, 18]
	EE-2	AGTCACTGACCCAACCTTAAATGGCTG			
	SSAP2f	GCTGAATGTGGGGATAATTTAT	55	641	
	SSAP2r	ATGGCTGCTTCCTTTCGGTTA			
*groEL*	EphplgroEL(569)F	ATGGTATGCAGTTTGATCGC	62	624	[19]
	EphplgroEL(1193)R	TCTACTCTGTCTTTGCGTTC			
	EphplgroEL(569)F	ATGGTATGCAGTTTGATCGC	56	573	
	EphgroEL(1142)R	TTGAGTACAGCAACACCACCGGAA			

### DNA sequencing and phylogenetic analysis

The amplified PCR products were purified using the TaKaRa Agarose Gel DNA Purification Kit Ver.2.0 (TaKaRa), ligated into pGEM-T Easy vector (Promega, Madison, WI, USA) and transformed into *Escherichia coli* JM109 competent cells (TaKaRa). Two recombinant clones were selected for sequencing using BigDye Terminator Mix (Sangon, Shanghai, China). The obtained sequences were analysed by a BLASTn search in GenBank or by using the ClustalW method in the MegAlign software (DNAStar, Madison, WI, USA). Phylogenetic trees were constructed based on the sequence distance method using the neighbor-joining (NJ) algorithm with the Kimura two-parameter model of the Mega 4.0 software [20].

### Statistical analysis

Statistical analysis was conducted using a Chi-square test in Predictive for Analytics Software Statistics 18 (PASW, SPSS Inc., Chicago, IL, USA). *P*-values of 0.05 or less were considered statistically significant.

### Nucleotide sequence accession numbers

The representative sequences obtained in this study have been submitted and deposited in the GenBank database with accession numbers KX272641–KX272643 for 16S rRNA and KX276166–KX276167 for *groEL* partial sequences.

## Results

Out of 421 sampled animals, 106 (25.2%) were positive for *A. phagocytophilum* (Table 2). The positive rates for *A. phagocytophilum* at different sampling sites varied from 0 to 75%. The positive rate was higher in sheep (35.1%, 40/114) than in goats (26.4%, 66/307) (*χ* ^2^ = 11.090, *df* = 1, *P* < 0.05). As shown in Table 2, the agent was detected in fourteen out of seventeen study sites in central and southeastern China.

**Table 2:** Detection of *A. phagocytophilum* in sheep and goats

Location		Species	No. infected/(%)		16S rRNA variant
Province	County		No. tested	No. positive (%)	
Chongqing	Wanzhou	Goat	24	7 (29.2)	variant 1
	Jiangjin	Goat	30	0 (0)	–
Guangxi	Pingxiang	Goat	11	0 (0)	–
	Jingxi	Goat	19	2 (10.5)	variant 1
Guizhou	Dushan	Goat	17	1 (5.9)	variant 1
	Rongjiang	Goat	25	6 (24)	variant 1
Hebei	Wangdu	Sheep	19	0 (0)	–
Hainan	Haikou	Goat	28	4 (14.3)	variant 1, 2
Sichuan	Hejiang	Goat	32	18 (56.3)	variant 1
	Panzhihua	Goat	32	1 (3.1)	variant 1
Shanxi	Lvliang	Sheep	50	15 (30.0)	variant 1, 2
Guangdong	Qingyuan	Goat	30	6 (20.0)	variant 1
	Zhaoqing	Goat	33	13 (39.4)	variant 1
Yunnan	Ruili	Goat	4	3 (75.0)	variant 1
	Fuyuan	Goat	7	2 (28.6)	variant 1
	Yanshan	Goat	15	3 (20.0)	variant 1
Hubei	Suizhou	Sheep	45	25 (55.6)	variant 1
Total			421	106 (25.2)	

**Fig. 1:**
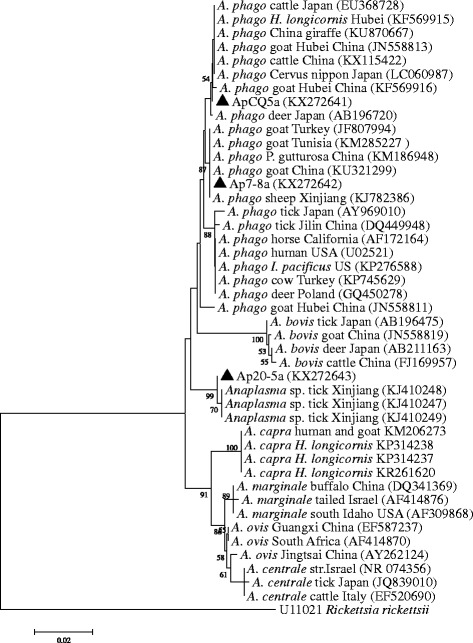
Phylogenetic analysis of *Anaplasma* spp. based on partial 16S rRNA gene sequences. Triangles indicate the sequences obtained in this study. *Abbreviations*: A. phago, *Anaplasma phagocytophilum*

The molecular characterization of *A. phagocytophilum* isolates in sheep and goats was analysed based on 16S rRNA and *groEL* genes. Forty-nine 16S rRNA sequences of *A. phagocytophilum* representative of different geographical locations were obtained in this study. The similarity among 16S rRNA gene sequences ranged from 99.7 to 100%. Phylogenetic analysis revealed that the *A. phagocytophilum* isolates identified in this study are placed in two separate clades (Fig. 1). Group 1, which contained 45 sequences (GenBank accession no. KX272641), was identical to the following strains: giraffe 2013-6, HB-C3, s4 and sika28, which were detected in the South African giraffe, *Haemaphysalis longicornis*, cattle and *Cervus Nippon* (GenBank accession nos. KU870667, KF569915, KX115422 and LC060987), respectively. Group 2 contained four sequences (GenBank accession nos. KX272642) and had 100% identity to the ApGGo2, Ap-SHAX31 and EKY155 strains derived from goats (GenBank accession nos. KM285227, KU321299 and JF807994), ATS1 from sheep (GenBank accession no. KJ782386) and JC3-3 from *Procapra gutturosa* (GenBank accession no. KM186948).

**Fig. 2:**
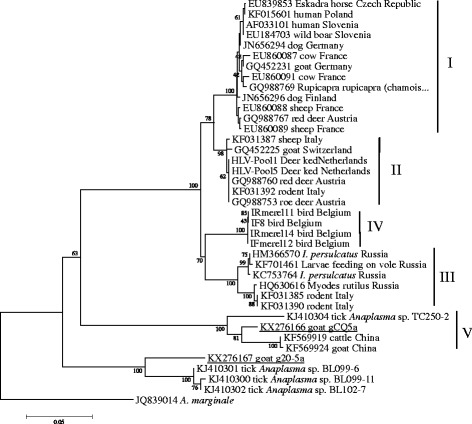
Phylogenetic analysis of *A. phagocytophilum* strains based on partial *groEL* gene sequences. The sequences from this study are underlined

Twenty-two *groEL* sequences obtained from the 16S rRNA gene positive samples were identical to each other and showed 96.1% identity to strain HB-MC-A25 of *A. phagocytophilum* from cattle (GenBank accession no. KF569919). Phylogenetic analysis was conducted with the *groEL* sequences in this study and sequences identified in different hosts and countries deposited in GenBank (*groEL* sequences are available in Additional file 1: Table S1). Five major *A. phagocytophilum* clusters could be proposed (labelled I-V in Fig. 2). The *groEL* sequences in this study formed one cluster (V), which was separate from the other four clusters or ecotypes reported in Europe. In addition, one isolate (20-5a) identified in a goat was different from known *Anaplasma* species (Figs. 1 and 2). The 16S rRNA and *groEL* sequences (GenBank accession nos. KX272643 and KX276167) showed 99.8 and 92.6% identity to the strains BL102-7 of an unclassified *Anaplasma* species from *Hyalomma asiaticum* ticks, respectively (GenBank accession nos. KJ410249 and KJ410302). The isolate was closely related to a novel *Anaplasma* sp. and clustered independently from all known *Anaplasma* species (Figs. 1 and 2).

## Discussion

*Anaplasma phagocytophilum* is an emerging tick-borne pathogen with veterinary and public health significance [1]. Since the first description of the agent in 1932, many wild and domestic animals have been considered as the reservoir hosts for *A. phagocytophilum* [1, 21]. In China, this species has been reported in sheep, goats, cattle, deer, yaks, dogs, rabbits and rodents in several provinces, and the infection rates vary in different hosts and/or geographical locations [14–16, 22, 23]. In this study, a molecular survey of *A. phagocytophilum* was conducted in sheep and goats in ten provinces in central and southeastern China. The positive rates of *A. phagocytophilum* in sheep and goats (25.2%, 106/421) was higher than those in goats from Jilin (5.7%, 8/35), Henan (13.0%, 6/46) and Hubei (14.5%, 10/69), and were lower than in sheep and goats (40.0%, 56/140) conducted in Gansu Province [13, 14, 16]. However, it is almost comparable with 26.69% prevalence in goats (126/472) shown in a previous report [24]. The positive rates for *A. phagocytophilum* were variable in sheep and goats as well as different sampling sites in China, and this may be related to sampling time and methods, tick vectors and methodical approaches. *Anaplasma phagocytophilum* infection was found in 14 of 17 study sites in the present study, implying an extensive geographical distribution of the agent in China. It is well known that *A. phagocytophilum* could cause a persistent infection in sheep and rodents, which allows them to be reservoirs of infection [25]. The sheep and goats serve as reservoir hosts may facilitate further spread of infection.

*Anaplasma phagocytophilum* exhibits a high degree of genetic diversity, host tropisms and variation in pathogenicity [7]. Genetic diversity of the agent has been reported in different hosts or geographical locations based on *groEL*, *ankA* and *msp4* [8–10]. Although high similarities of the 16S rRNA gene sequences were reported, several 16S rRNA gene variants have been identified [17, 26]. In this study, two 16S rRNA variants of *A. phagocytophilum* were identified in sheep and goats. Phylogenetic analysis revealed that variant 1 (GenBank accession no. KX272641) clustered, with 100% identity, together with the strains isolated from cattle, giraffe, goats and *H. longicornis* in China, sika deer (*Cervus nippon*) and cattle in Japan (Fig. 1), indicating that it circulates in a wide range of vertebrate hosts in Asia. Variant 2 (GenBank accession no. KX272642) has been detected in sheep, goats and Mongolian gazelle (*Procapra gutturosa*) in China and goats in Tunisia and Turkey (Fig. 1), implying that it is distributed worldwide. Moreover, variant 1 (91.8%) appears to be the most dominant strain in sheep and goats in China.

In accordance with the existing genetic diversity at the level of nucleotide sequences of the *groEL* gene, it has been used for the distinction of lineages or ecotypes represented by a variety of isolates in the environment and clinical samples [9, 11, 26]. A previous report has revealed that *A. phagocytophilum* circulates in Eastern Europe and belongs to two *groE*S*L* lineages and the strains pathogenic for humans and animals were clustered within lineage one [26]. Recently, four geographically dispersed ecotypes with different host ranges and zoonotic potential were identified based on *groESL* in Europe [3]. In the present study, the *groEL* sequences of *A. phagocytophilum* in sheep and goats formed one cluster (ecotype V), which was distinct from the known ecotypes in Europe (Fig. 2). According to the previous reports, *A. phagocytophilum* strains within ecotype I had a wide host range and were commonly isolated from wild and domestic animals as well as humans [3]. Strains that are pathogenic to humans and animals were found within ecotype I, indicating the pathogenic potential of members in this ecotype. Ecotype II was mainly found in roe deer and rodents, ecotype III in rodents and ecotype IV in birds (Fig. 2) [3]. Clinical signs in wild species are very difficult to observe. Little is known about pathogenicity of the different variants of *A. phagocytophilum* in these species. Ecotype V has been found not only in sheep and goats in this study, but also in ticks and cattle in previous reports (Figs. 1 and 2) [27], suggesting the circulation and wide distribution of this ecotype in China. However, very limited clinical data are available so far, and it is unknown whether the detected strains in ecotype V are pathogenic to humans or livestock animals in China. Clearly, the zoonotic potential of the members within ecotype V need to be further investigated.

Currently, the recognized *Anaplasma* species include *A. phagocytophilum*, *A. marginale*, *A. centrale* (*A. marginale* subsp. *centrale*), *A. ovis*, *A. bovis* and *A. platys*. In addition to the above mentioned species, a novel *Anaplasma* species designated “*Anaplasma capra*” has been identified in goats, ticks and humans in northern China [28]. In addition to well-characterized *Anaplasma* species, a number of new *Anaplasma* genetic variants have been identified increasingly in ticks and vertebrates using molecular techniques, especially in wildlife [7, 17, 29, 30]. In this study, a potential novel *Anaplasma* species was identified in goat. This isolate was genetically distinct from other known *Anaplasma* species based on 16S rRNA and *groEL* genes and is closely related to an unclassified *Anaplasma* species, which has been identified in *H. asiaticum* ticks from Xinjiang, northwestern China [27]. The global health burden and risk of anaplasmosis on human and animals seems to be underestimated [31], we may assume that additional novel *Anaplasma* species remain undiscovered and contribute to human and/or animal diseases. Further investigation is needed with respect to the effects of anaplasmoses on both human and animals.

## Conclusions

A molecular survey of *A. phagocytophilum* was conducted in ruminants from ten provinces in central and southeastern China. *Anaplasma phagocytophilum* was detected in 106 (25.2%) sheep and goats. Two 16S rRNA variants and a new ecotype (V) of *A. phagocytophilum* were identified and endemic in small ruminants in China. Moreover, a potential novel *Anaplasma* species was detected in goat in the present study. Our findings might provide valuable information for the control and management of anaplasmosis in China.

## Additional file

Additional file 1: Table S1.
*GroEL* gene sequences of *A. phagocytophilum* isolates analysed in this study. (XLSX 19 kb)

## Abbreviations

DNA: Deoxyribonucleic acid
EDTA: Ethylene diamine tetraacetic acid
*groEL*: heat-shock operon
HGA: Human granulocytic anaplasmosis
PCR: Polymerase chain reaction
UV: Ultraviolet.
